# Community-based health-focused longitudinal aging studies in East and Southeast Asia: landscape and future directions

**DOI:** 10.1016/j.lanwpc.2026.101867

**Published:** 2026-05-02

**Authors:** Rahul Malhotra, Hanzhang Xu, Haolin Li, Meagan Goh Yijing, Leila Marie Magno, Huashuai Chen, Dararatt Anantanasuwong, Hideki Hashimoto, Zachary Zimmer, Halimah Awang, Yaohui Zhao, Vivian W.Q. Lou, Wenqian Xu, Yunhwan Lee, Lijing L. Yan, Grace T. Cruz, Chenkai Wu

**Affiliations:** a Centre for Ageing Research & Education, Duke-NUS Medical School, Singapore; b Health Services Research & Population Health, Duke-NUS Medical School, Singapore; c School of Nursing, Duke University, Durham, NC, USA; d Department of Family Medicine and Community Health, School of Medicine, Duke University, Durham, NC, USA; e Global Health Research Center, Duke Kunshan University, Kunshan, Jiangsu, China; f University of the Philippines Population Institute, Quezon City, Philippines; g Business School, Xiangtan University, Xiangtan, Hunan, China; h Center for Aging Society Research at National Institute of Development Administration, Bangkok, Thailand; i Department of Health and Social Behavior, School of Public Health, University of Tokyo, Tokyo, Japan; j Department of Aging and Family Science, Mount Saint Vincent University, Halifax, Canada; k Social Wellbeing Research Centre, Universiti Malaya, Kuala Lumpur, Malaysia; l Dong Fureng Institute of Economic and Social Development, Wuhan University, Wuhan, Hubei, China; m Department of Social Work and Social Administration, University of Hong Kong, Hong Kong, China; n Sau Po Centre of Ageing, University of Hong Kong, Hong Kong, China; o Department of Health Sciences, Lund University, Lund, Sweden; p Department of Preventive Medicine and Public Health, Ajou University School of Medicine, Suwon, Republic of Korea; q Institute on Aging, Ajou University Medical Center, Suwon, Republic of Korea

**Keywords:** Healthy aging, Longitudinal studies, East Asia, Southeast Asia, Health Data

## Abstract

East and Southeast Asia's (ESEA) rapidly aging population creates an urgent need for comprehensive health data to inform policies promoting healthy aging. However, a synthesis of the region's community-based longitudinal aging studies is lacking. We conducted a systematic search for community-based longitudinal aging studies in ESEA. Studies were included if they followed community-dwelling adults with a mean age of 50+ at baseline, had nationally representative samples or sampled from at least two geographic areas, were health focused, and offered English documentation. We identified 30 eligible studies across 10 countries, regions, or territories, mostly concentrated in Japan, Mainland China, Singapore, and South Korea. While recent studies incorporate biomarkers and performance measurements, significant gaps were found. No eligible studies exist for several countries (e.g., Myanmar, Cambodia, and Indonesia), physical frailty is rarely assessed, data access is inconsistent, and no multi-national studies were identified. Although data infrastructure for longitudinal aging studies exists in ESEA, there are critical gaps in geographic representation, measurement harmonization, and data access. Future efforts must enhance regional coordination, standardize core measures, and improve data-sharing mechanisms to promote and support healthy aging in ESEA.

## Introduction

**Fig. 1:**
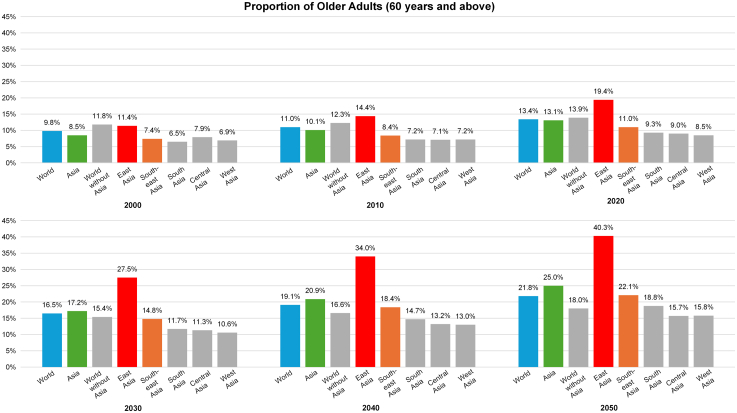
Proportion of Older Adults (Aged 60 Years and Above) in the Population: World, Asia, the World without Asia, and the Asian Region (East Asia, Southeast Asia, South Asia, Central Asia, West Asia). East Asia: Mainland China, Hong Kong SAR, Democratic People's Republic of Korea (North Korea), Japan, Macao SAR, Mongolia, Republic of Korea (South Korea), Taiwan. Central Asia: Kazakhstan, Kyrgyzstan, Tajikistan, Turkmenistan, Uzbekistan. South Asia: Afghanistan, Bangladesh, Bhutan, India, Iran (Islamic Republic of), Maldives, Nepal, Pakistan, Sri Lanka. Southeast Asia: Brunei Darussalam, Cambodia, Indonesia, Lao People's Democratic Republic, Malaysia, Myanmar, the Philippines, Singapore, Thailand, Timor-Leste, Vietnam. West Asia: Armenia, Azerbaijan, Bahrain, Cyprus, Georgia, Iraq, Israel, Jordan, Kuwait, Lebanon, Oman, Qatar, Saudi Arabia, State of Palestine, Syrian Arab Republic, Türkiye, United Arab Emirates, Yemen.

**Fig. 2:**
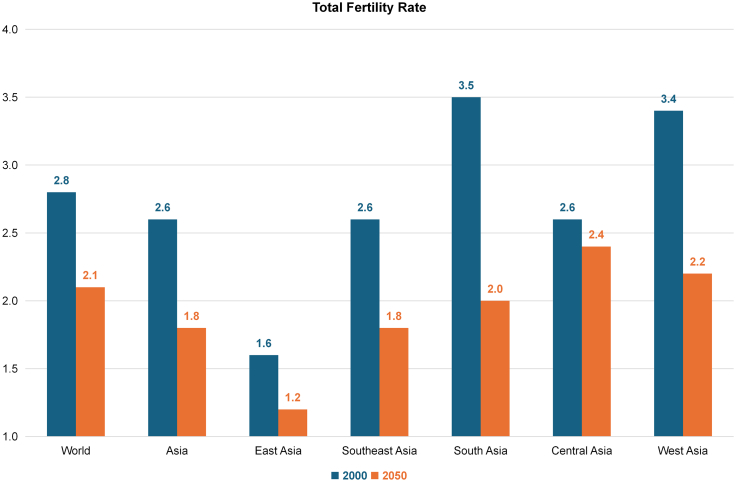
Total Fertility Rate from 2000 to 2050: World, Asia, East Asia, Southeast Asia, West Asia, Central Asia, South Asia. East Asia: Mainland China, Hong Kong SAR, Democratic People's Republic of Korea (North Korea), Japan, Macao SAR, Mongolia, Republic of Korea (South Korea), Taiwan. Central Asia: Kazakhstan, Kyrgyzstan, Tajikistan, Turkmenistan, Uzbekistan. South Asia: Afghanistan, Bangladesh, Bhutan, India, Iran (Islamic Republic of), Maldives, Nepal, Pakistan, Sri Lanka. Southeast Asia: Brunei Darussalam, Cambodia, Indonesia, Lao People's Democratic Republic, Malaysia, Myanmar, the Philippines, Singapore, Thailand, Timor-Leste, Vietnam. West Asia: Armenia, Azerbaijan, Bahrain, Cyprus, Georgia, Iraq, Israel, Jordan, Kuwait, Lebanon, Oman, Qatar, Saudi Arabia, State of Palestine, Syrian Arab Republic, Türkiye, United Arab Emirates, Yemen.

**Fig. 3:**
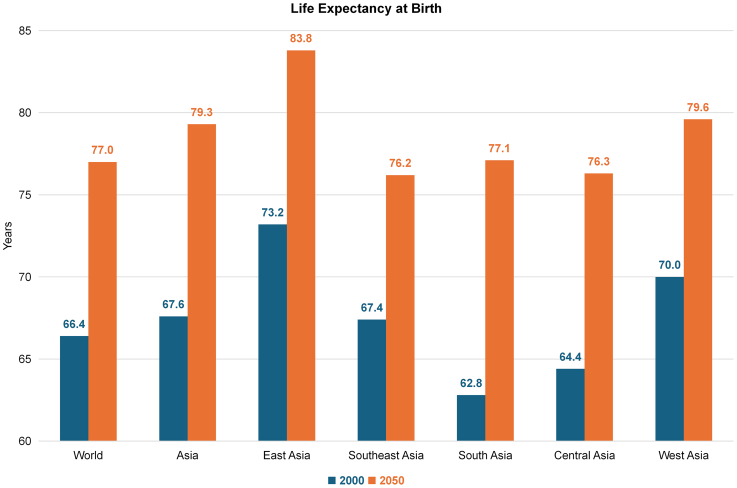
Life Expectancy at Birth from 2000 to 2050: World, Asia, East Asia, Southeast Asia, West Asia, Central Asia, South Asia. East Asia: Mainland China, Hong Kong SAR, Democratic People's Republic of Korea (North Korea), Japan, Macao SAR, Mongolia, Republic of Korea (South Korea), Taiwan. Central Asia: Kazakhstan, Kyrgyzstan, Tajikistan, Turkmenistan, Uzbekistan. South Asia: Afghanistan, Bangladesh, Bhutan, India, Iran (Islamic Republic of), Maldives, Nepal, Pakistan, Sri Lanka. Southeast Asia: Brunei Darussalam, Cambodia, Indonesia, Lao People's Democratic Republic, Malaysia, Myanmar, the Philippines, Singapore, Thailand, Timor-Leste, Vietnam. West Asia: Armenia, Azerbaijan, Bahrain, Cyprus, Georgia, Iraq, Israel, Jordan, Kuwait, Lebanon, Oman, Qatar, Saudi Arabia, State of Palestine, Syrian Arab Republic, Türkiye, United Arab Emirates, Yemen.

Projections from the United Nations indicate a substantial surge in the number of older adults (i.e., individuals aged 60 years and older) globally, reaching 1.4 billion by the end of this decade and 2.1 billion by 2050.^1^ This demographic shift is particularly pronounced in Asia, where the proportion of older adults is increasing more rapidly than in the rest of the world (Fig. 1).^1^ Within Asia, East and Southeast Asia (ESEA) are at the forefront of this demographic transition, largely driven by declining fertility rates and increasing life expectancy (Figs. 2 and 3).^1^

This increasing proportion of older adults in ESEA is linked to a rising burden of age-related diseases, particularly non-communicable diseases (NCDs).^2^, ^3^, ^4^ In 2019, NCDs accounted for most of the disease burden in older adults in East Asia (92.8%) and Southeast Asia (86.3%).^2^^,^^5^ This trend is accompanied by a growing prevalence of dementia, mental health conditions, and limitations in activities of daily living (ADLs) and instrumental ADLs (IADLs).^6^^,^^7^ Consequently, there is an urgent need for comprehensive health-focused data on older adults in ESEA to enable effective monitoring and surveillance of health trends and understand the current situation and regional variations in aging-related health outcomes. Such data is essential for developing and refining evidence-based interventions and social- and health-related policies for promoting healthy aging.

Organizations such as the World Health Organization (WHO)^8^ and World Economic Forum^9^ advocate for quality data to not only facilitate statistical analysis but also foster interdisciplinary research that strengthens health systems and healthcare delivery for older adults. While sociodemographic and health-focused data of older adults are currently available from certain nations (e.g., Mainland China, Japan, Singapore, and Thailand), they remain largely limited in many other countries, regions, or territories in ESEA.^10^ Not surprisingly, the United Nations Decade of Healthy Aging (2021–2030) identifies the collection of high-quality, global data and the promotion of research on healthy aging as a key objective.^10^

A crucial determinant of high-quality data is sample representativeness. Representative samples enable unbiased evaluation of the relationships between confounders, exposures, and outcomes, even for variables not specified in the original hypotheses.^11^ Given that most of older adults in ESEA are community-dwelling,^12^ community-based studies of aging cohorts, particularly when representative of a defined population, allow for the accurate estimation of distributions, prevalences, and risk factor trends. Consequently, representative community-based studies offer higher external validity and generalizability than other data sources.^13^

Longitudinal (or cohort) studies, especially when combined with representative sampling strategies, are key tools to advance our understanding of the aging process.^14^, ^15^, ^16^, ^17^ Unlike cross-sectional studies, which provide a snapshot in time, longitudinal studies allow researchers to examine how health and disease evolve within individuals over time. This design provides a clearer understanding of aging trajectories, captures period and cohort effects, and enables between-individual comparisons to identify similarities and differences in aging patterns.^14^, ^15^, ^16^, ^17^ Longitudinal data can also establish temporal relationships between exposures and outcomes, which is essential for causal inference.^15^, ^16^, ^17^ By capturing the dynamic nature of the aging processes, including transitions between health states (e.g., from pre-frail to frail or vice-versa) and the accumulation of multiple chronic conditions, longitudinal studies highlight the holistic and interdisciplinary nature of aging research and facilitate data harmonization and cross-national comparisons.^17^

Despite the established importance of longitudinal health-focused aging research, there remains a significant knowledge gap in understanding the landscape of available longitudinal health-focused aging studies in ESEA. Currently, there is no systematic synthesis of such studies in the region, limiting researchers' and policymakers' ability to identify existing resources and gaps in data collection and access. This lack of comprehensive overview may also limit effective utilization of available data and coordination of research efforts in ESEA across countries, regions or territories with diverse socio-economic and cultural contexts. Given the rapid increase in the older adult population in ESEA, the region will benefit from enhanced coordination in generating evidence-based solutions through strategic local and regional data collection and harmonization initiatives.^18^ Such coordinated efforts are crucial for developing contextually appropriate interventions and policies that address the unique challenges of population aging in Asia. The current situation presents an opportunity for increased regional collaboration and investment in local research infrastructure to systematically map existing data sources and identify priority areas for future data collection efforts.

This paper maps the current landscape of community-based health-focused longitudinal aging studies in ESEA, synthesizing their methodologies and data resources, and identifying research gaps. It contributes to the discourse on aging research in ESEA, and globally, by addressing the challenges posed by recent global policy shifts to international collaborations and research funding, which underscores the need for regional collaboration and self-reliance. By raising awareness of these valuable data resources, this paper aims to promote the generation of locally relevant evidence to inform policies and interventions in ESEA for improving healthy aging and facilitating cross-national comparisons within this rapidly aging and diverse region.

## Methods

### Search strategies

As in prior research,^19^^,^^20^ we conducted a systematic search to identify community-based health-focused longitudinal aging studies conducted in ESEA using the following data archives: National Archive of Computerized Data on Aging, Integrative Analysis of Longitudinal Studies on Aging, Gateway to Global Aging Data, and WHO Multi-Country Studies Data Archive. We also searched PubMed for articles that described longitudinal aging studies in ESEA. Furthermore, we searched for relevant articles in the International Journal of Epidemiology and BMJ Open as these two journals have a specific section dedicated to cohort profile papers. Key search terms used in each data source are presented in Supplementary Table S1. Lastly, we conducted a manual search of reference lists of identified articles and contacted experts in the field to identify any additional studies that may not have been captured through the search strategies described above.

We restricted our search to data archive entries or articles published between 2000 and 2024 to capture longitudinal studies that reflect more contemporary aging experiences in ESEA. The lower bound of 2000 was selected to ensure that included datasets reflect modern health systems, social structures, and data collection practices. However, we included longitudinal studies that were initiated prior to 2000 if they had any waves of data collection between 2000 and 2024. The upper bound of 2024 corresponds to the most recent complete publication year prior to our search date (February 20, 2025), as articles published in 2025 may not yet be fully indexed or assigned a definitive publication year in bibliographic databases.

### Inclusion and exclusion criteria

**Fig. 4:**
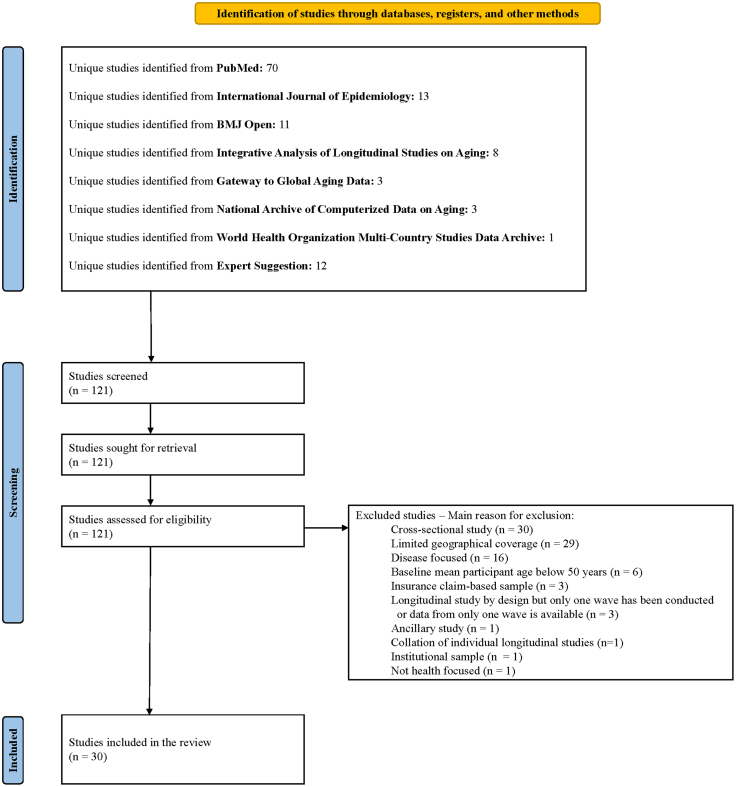
Selection process of included studies.

Studies were included if they met *all* of the following criteria, based on published data: (1) conducted in ESEA countries, regions or territories, (2) had a longitudinal design with data from at least one follow-up wave after baseline available, (3) included community-dwelling adults with mean age at baseline of 50 years or older, (4) included a nationally representative sample by study design or sampled from at least two geographic areas (e.g., provinces, states, or planning areas), (5) was health-focused, and (6) had documentation of study methodology and cohort baseline characteristics available in English or a local language with an English abstract. In addition to studies that did not meet the inclusion criteria, we also excluded longitudinal studies that: (1) exclusively recruited individuals from institutional settings or insurance claim datasets, (2) were limited to patient populations with a specific disease or condition (e.g., those with diabetes), or (3) were ancillary studies of an included study. We also excluded any collations of individual longitudinal studies. The selection process is presented in Fig. 4. Main reasons for exclusion are presented in Supplementary Table S2 for all ineligible studies.

### Data extraction and analysis

**Fig. 5:**
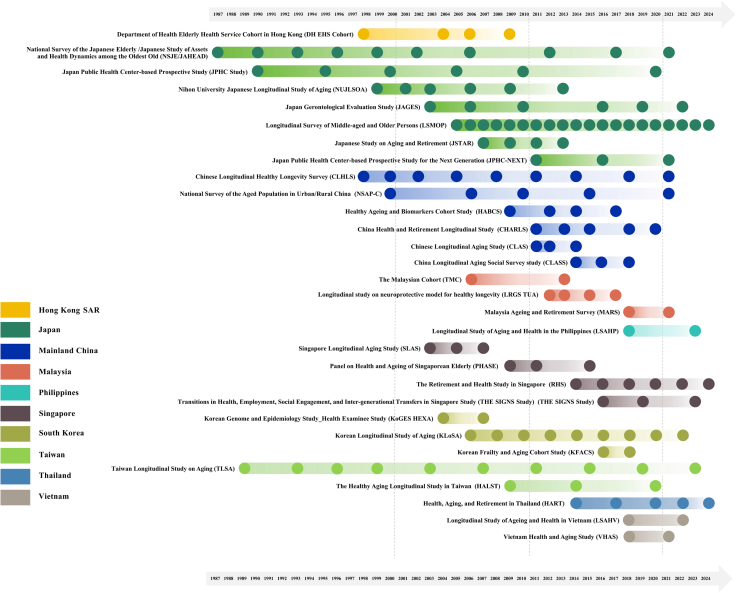
Timeline of included studies.

Two independent reviewers (MGY and LMM) screened the titles and abstracts or summary descriptions of all retrieved records and then reviewed the documentation of potentially eligible studies to determine eligibility. Any discrepancies in the selection process were discussed with the larger team to reach consensus. For each included study, the independent reviewers extracted the following information: study name; country, region or territory; baseline and follow-up wave years; sample size per wave; data collection methods; participant characteristics at baseline (age in years [mean and range], gender distribution, and education categories); data access and sharing policies; and health-specific constructs measured (e.g., physical function, cognitive function, social connectedness, anthropometric variables). One reviewer (MGY or LMM) extracted these data elements into a standardized Excel spreadsheet and then a second reviewer (HL) validated each record. We conducted a narrative synthesis of the identified longitudinal studies, focusing on their populations studied, methodological approaches, and key measurement constructs. We also created a visualization for the timeline of the included studies (Fig. 5).

## Results

### Setting

Of the 121 studies yielded from our search, we included 30 eligible community-based health-focused longitudinal aging studies in ESEA (Fig. 4). These studies collectively represented 10 countries, regions, or territories, with Japan (7 studies), Mainland China (6 studies), Singapore (4 studies), Malaysia (3 studies), and South Korea (3 studies) having the most representation. Taiwan and Vietnam each had two longitudinal studies, while Hong Kong SAR, the Philippines, and Thailand each had one longitudinal study. None of the studies included samples from more than one country, region, or territory (Fig. 5).

### Temporal distribution

The earliest longitudinal studies began in the late 1980s, with two cohorts initiated between 1987 and 1989: the Taiwan Longitudinal Study on Aging (TLSA)^21^ and the National Survey of the Japanese Elderly (NSJE)/Japanese Study of Assets and Health Dynamics among the Oldest Old (JAHEAD).^22^ Following these early efforts, four additional cohorts were established in the 1990s, including the Chinese Longitudinal Healthy Longevity Survey (CLHLS) in Mainland China,^23^ the Department of Health Elderly Health Service Cohort in Hong Kong SAR,^24^ Japan Public Health Center-based Prospective Study (JPHC Study),^25^ and the Nihon University Japanese Longitudinal Study of Aging (NUJLSOA).^26^ A significant expansion occurred in the 2000s, with 24 new longitudinal studies launched across ESEA.^27^, ^28^, ^29^, ^30^, ^31^, ^32^, ^33^, ^34^, ^35^, ^36^, ^37^ Among which, 13 studies were initiated between 2010 and 2019.^38^, ^39^, ^40^, ^41^, ^42^, ^43^, ^44^, ^45^, ^46^, ^47^, ^48^, ^49^, ^50^ However, we did not identify any new longitudinal study launched after 2020 (Fig. 5).

Most studies have been collecting follow-up data every 2–4 years. As shown in Fig. 5, currently, only the Longitudinal Survey of Middle-aged and Older Persons (LSMOP) in Japan,^30^ has follow-up periods spanning more than a decade with annual follow-ups.

### Data collection methods

The most common data collection methods (not mutually exclusive) employed in the studies were face-to-face interviews (n = 28 studies), anthropometric measurement (e.g., height, weight, waist circumference, body fat etc.; n = 22), blood pressure measurement (n = 20), physical performance measurement (e.g., handgrip strength, gait speed etc.; n = 19), biospecimen collection (e.g., blood, hair etc.; n = 14), and physical/clinical examination (n = 11, Table 1). Less common methods included administrative data linkage, telephone interviews, mail surveys and leave-behind questionnaires. Most studies (n = 28) adopted more than one data collection method. Furthermore, newer studies and/or more recent waves of data collection from existing studies were more likely to employ multiple data collection methods, alongside the inclusion of biospecimen collection, physical performance measurement and physical/clinical examination.

**Table 1:** Key information and sample descriptive statistics of included studies (n = 30).

S. No.	Name of study	Country/Region/Territory	Baseline (Wave 1) year	Follow-up years (Wave: Year)	Sample size (Wave)	Data collection methods	Key sample descriptives statistics, at baseline					Data access and sharing
							Age range (years)	Mean age (years)	% aged ≥60 years	Sex (% men)	Education categories (as categorized in the study)	
1	Department of Health Elderly Health Service Cohort in Hong Kong	Hong Kong SAR	1999–2001	Wave 2: 2004 Wave 3: 2006 Wave 4: 2009	66,820 (Wave 1) 12,958 (Wave 2) 2441 (Wave 3) 3996 (Wave 4)	*Baseline:* Face-to-face interview (unspecified^a^) Anthropometric measurements Biospecimen collection Blood pressure Physical/clinical examination *Follow-up:* Administrative data (link with death registration and public healthcare services) Telephone interview	65 years and above	72.4	100.0%	34.0%	–c	No plans for public data access/usage in the immediate future. Further details may be obtained from hkusph@hku.hk. The study team, however, are keen to obtain full value from the cohorts and would welcome proposals to exploit this cohort from potential collaborators or from researchers interested in investigating specific questions independently.
2	Japan Gerontological Evaluation Study (JAGES)	Japan	2003–2004	Wave 2: 2006–2007 Wave 3: 2010–2011 Wave 4: 2013 Wave 5: 2016–2017 Wave 6: 2019–2020 Wave 7: 2022–2023	32,891 (Wave 1) 39,765 (Wave 2) 138,000 (Wave 3) 153,468 (Wave 4) 205,772 (Wave 5) 266,113 (Wave 6) 228,528 (Wave 7)	Mail survey	65 years and above	–	100.0%	–	–	Collected data is available for open access after the submission of a proposal online and adherence to other protocols. Details of requests can be found on the JAGES website (https://www.jages.net/data_application/).
3	Japan Public Health Center-based Prospective Study (JPHC Study)	Japan	1990 (Cohort I) 1993 (Cohort II)	Wave 2: 1995 & 1998 Wave 3: 2000 & 2003 Wave 4: 2005 & 2008 Wave 5: 2010 & 2013 Wave 6: 2020 & 2023 (planned)	113, 461 (Wave 1) 103,734 (Wave 2) 99,498 (Wave 3)	*Baseline:* Mail survey Anthropometric measurements Biospecimen collection Physical/clinical examination Telephone interview *Follow-up:* Administrative data	Cohort I: 40–59 years Cohort II: 40–69 years	–	–	47.0%	–	Investigators are granted access to JPHC data and/or biospecimens as collaborative study after approval of the JPHC Steering Committee (SC) and the Institutional Review Board (IRB). Currently only citizens of Japan who fulfill the requirements of conducting research projects are eligible to apply for the JPHC data and/or biospecimens. Investigators are required to submit a Project Protocol (Research question, Aim, Background, Design and analytical plan) for review by the JPHC SC. Requests can be made by contacting the JPHC SC directly (jphcadmin@ml.res.ncc.go.jp) or investigators in JPHC (Dr Shoichiro Tsugane or Dr Norie Sawada). More information can be found on the website (https://epi.ncc.go.jp/en/jphc/805/index.html).
4	Japan Public Health Center-based Prospective Study for the Next Generation (JPHC-NEXT)	Japan	2011–2016	Wave 2: 2016–2021 Wave 3: 2021-ongoing Follow-up planned every 5 years	115,385 (Wave 1) 91,404 (Wave 2)	*Baseline:* Mail survey Biospecimen collection Blood pressure Face-to-face interview (unspecified) Telephone interview *Follow-up:* Administrative data	40–74 years	–	51.4%	46.1%	Secondary school: 19.5% High school: 50.2% Vocational school/junior college/college dropout: 12.9% University and above: 16.5%	Currently only citizens of Japan who fulfill the requirements of conducting research projects are eligible to apply for the JPHC-NEXT data and/or biospecimens. Investigators are required to submit a Project Protocol (Research question, Aim, Background, Design and analytical plan) for review by the JPHC-NEXT SC. Requests can be made by contacting the JPHC-NEXT SC directly (jphcadmin@ml.res.ncc.go.jp) or investigators in JPHC-NEXT (Dr Shoichiro Tsugane or Dr Norie Sawada). More information can be found on the website (https://epi.ncc.go.jp/jphcnext/en/access/index.html).
5	Japanese Study on Aging and Retirement (JSTAR)	Japan	2007	Wave 2: 2009 Wave 3: 2011 Wave 4: 2013	4163 (Wave 1) 4657 (Wave 2) 5759 (Wave 3) 4545 (Wave 4)	Face-to-face interview (CAPI^b^) Anthropometric measurements Blood pressure Leave-behind questionnaire Physical performance measurement	50–75 years	63.4	61.4%	48.0%	Less than high school: 24.7% High school: 44.1% Vocational/junior college: 13.8% College: 15.9% Graduate school (master/doctor): 1.0%	Data will be made available free of charge, but only to qualified researchers, higher education institutes, and administrative agencies that have agreed to follow the stringent conditions of use set by the Research Institute of Economy, Trade and Industry and only in the case that such data are used for academic and statistical research purposes. Approval or disapproval of applications for the use of JSTAR data will be determined by RIETI after considering the opinion of an internal advisory committee, and notification will be sent to applicants. Details can be found at: https://www.rieti.go.jp/en/projects/jstar/index.html
6	Longitudinal Survey of Middle-aged and Older Persons (LSMOP)	Japan	2005	Annually	34,240 (Wave 1) 32,285 (Wave 2) 30,730 (Wave 3) 29,605 (Wave 4) 28,736 (Wave 5) 26,220 (Wave 6) 25,321 (Wave 7) 24,026 (Wave 8) 23,722 (Wave 9) 22,748 (Wave 10) 22,595 (Wave 11) 21,916 (Wave 12) 21,168 (Wave 13) 20,677 (Wave 14) 19,931 (Wave 15) 19,644 (Wave 16) 18,999 (Wave 17) 18,469 (Wave 18) 17,875 (Wave 19)	Waves 1–5: Face-to-face interview (unspecified) Waves 6 onwards: Mail survey	50–59 years	46.0% aged 50–54 years, 54.0% aged 55–59 years	0.0%	48.5%	–	Data is publicly available across waves at: https://www.e-stat.go.jp/stat-search/files?page=1&toukei=00450045&tstat=000001030155
7	National Survey of the Japanese Elderly (NSJE)/Japanese Study of Assets and Health Dynamics among the Oldest Old (JAHEAD)	Japan	1987	Wave 2: 1990 Wave 3: 1993 Wave 4: 1996 Wave 5: 1999 Wave 6: 2002 Wave 7: 2006 Wave 8: 2012 Wave 9: 2017 Wave 10: 2021	2200 (Wave 1) 2227 (Wave 2) 2061 (Wave 3) 2751 (Wave 4) 3989 (Wave 5) 3245 (Wave 6) 2459 (Wave 7) 2940 (Wave 8) 1748 (Wave 9) 1933 (Wave 10)	Face-to-face interview (unspecified) Anthropometric measurements Leave-behind questionnaire Mail survey (Wave 10) Physical performance measurement	60 years and above	–	100.0%	–	–	Data is publicly available for the first eight waves at the study website (https://www2.tmig.or.jp/jahead/eng/index.html), although there may be eligibility requirements. Data can also be accessed at the Social Science Japan Data Archive of The University of Tokyo (http://csrda.iss.u-tokyo.ac.jp/) or the Inter-university Consortium for Political and Social Research (https://www.icpsr.umich.edu/web/pages/)
8	Nihon University Japanese Longitudinal Study of Aging (NUJLSOA)	Japan	1999	Wave 2: 2001 Wave 3: 2003 Wave 4: 2006 Wave 5: 2009 Wave 6: 2013	4997 (Wave 1) 4623 (Wave 2) 4507 (Wave 3) 3403 (Wave 4) 2575 (Wave 5) 1444 (Wave 6)	Face-to-face interview (unspecified) Anthropometric measurements Blood pressure Physical performance measurement	65 years and above	33.0% aged 65–69 years, 27.5% aged 70–74 years, 20.3% aged 75–79 years, 12.7% aged 80–84 years, 6.0% aged 85 years and above	100.0%	43.9%	–	Permission is required for the use of the data through an application form. Consent for usage will be determined by the study lead. More information can be obtained from Professor Yasuhiko Saito (saito.yasuhiko@nihon-u.ac.jp).
9	China Health and Retirement Longitudinal Study (CHARLS)	Mainland China	2011	Wave 2: 2013 Wave 3: 2015 Wave 4: 2018 Wave 5: 2020	17,697 (Wave 1) 18,254 (Wave 2) 20,273 (Wave 3) 19,816 (Wave 4) 19,395 (Wave 5)	Face-to-face interview (CAPI) Anthropometric measurements Biospecimen collection Blood pressure Physical/clinical examination Physical performance measurement	45 years and above	59.7	43.0%	47.9%	No formal education (36.5%), Incomplete primary school (18.8%), Completed primary school (20.3%), Completed middle school (12.8%), Completed high school (6.8%), College and above (2.6%) (For >60)	Data is publicly accessible but requires log-in credentials through the study website: https://charls.charlsdata.com/pages/data/111/en.html. More details can be found on the study website.
10	China Longitudinal Aging Social Survey study (CLASS)	Mainland China	2014	Wave 2: 2016 Wave 3: 2018 Wave 4: 2020	11,511 (Wave 1), not publicly available for Waves 2–4	Face-to-face interview (unspecified) Anthropometric measurements	60 years and above	70.7	100.0%	47.9%	Mean years of education: 13.0 ± 2.5	Documentation of the baseline study in 2014 is publicly accessible through the project website (http://class.ruc.edu.cn/English/Home.htm). Other project data requires log-in credentials in the webpage. Data from waves 2–3 can be downloaded from the Inter-university Consortium for Political and Social Research after logging in: https://www.openicpsr.org/openicpsr/project/191983/version/V1/view
11	Chinese Longitudinal Aging Study (CLAS)	Mainland China	2011	Wave 2: 2012 Wave 3: 2014	3514 (Wave 1), not publicly available for subsequent waves	Face-to-face interview (CAPI) Biospecimen collection Physical/clinical examination	60 years and above	72.8	100.0%	–	–	Data catalog of CLAS can be found at https://www.maelstrom-research.org/study/clas while details can be found at the DPAU Contributing Research Studies (CRS) Directory https://portal.dementiasplatform.com.au/crs-directory/clas
12	Chinese Longitudinal Healthy Longevity Survey (CLHLS)	Mainland China	1998	Wave 2: 2000 Wave 3: 2002 Wave 4: 2005 Wave 5: 2008–2009 Wave 6: 2011–2012 Wave 7: 2014 Wave 8: 2018 Wave 9: 2021	8959 (Wave 1) 11,161 (Wave 2) 20,428 (Wave 3) 18,549 (Wave 4) 20,366 (Wave 5) 10,190 (Wave 6) 7107 (2014, Wave 7), not publicly available for Wave 8–9	Face-to-face interview (unspecified) Anthropometric measurements Biospecimen collection Blood pressure Leave-behind questionnaire Physical performance measurement	80 years and above	92.3	100.0%	39.8%	–	Researchers can download unrestricted data and study documents from the Peking University Open Research Data Platform after registration and requests (https://opendata.pku.edu.cn/dataverse/CHARLS). Study documents, however, are publicly accessible.
13	Healthy Ageing and Biomarkers Cohort Study (HABCS)	Mainland China	2008	Wave 2: 2012 Wave 3: 2014 Wave 4: 2017	1462 (Wave 1) 2311 (Wave 2) 2493 (Wave 3) 2958 (Wave 4)	Face-to-face interview (unspecified) Anthropometric measurements Biospecimen collection Blood pressure Physical/clinical examination Physical performance measurement	65 years and above	88.0	100.0%	37.1%	Education ≥ 1 year (%): 67.3% for age 65–79, 34.2% for age 80–89, 21.8% for age 90–99, 8.7% for age 100 years and above	Publicly accessible but requires a letter of undertaking and application form to be submitted (Cohort_HABCS@163.com)
14	National Survey of the Aged Population in Urban/Rural China	Mainland China	2000	Wave 2: 2006 Wave 3: 2010 Wave 4: 2015 Wave 5: 2021	20,255 (Wave 1) 19,947 (Wave 2) 19,986 (Wave 3) 220,170 (Wave 4) 127,287 (Wave 5)	Face-to-face interview (unspecified)	60 years and above	–	100.0%	53.0%	Unschooled (42.5%), Primary school (34.3%), Junior high (11.4%), Senior high (7.0%), College and above (4.2%)	Data available upon request after signing data request form (miaowsh@163.com). More details can be found on the study website (http://www.crca.cn/index.php/76-data-resource/data-service/1116-2024-10-17-06-26-41.html).
15	Longitudinal Study on Neuroprotective Model for Healthy Longevity (LRGS TUA)	Malaysia	2012–13	Wave 2: 18 months post-baseline Wave 3: 36 months post-baseline Wave 4: 5 years post-baseline	2322 (Wave 1) 1480 (Wave 2) 1328 (Wave 3)	Face-to-face interview (CAPI) Anthropometric measurements Biospecimen collection Blood pressure Physical performance measurements	60 years and above	69.1	100.0%	48.0%	No formal education (21.2%), Primary education (57.6%) Secondary education (19.1%), Tertiary education (2.1%)	Data is accessible via a direct application to Dementias Platform Australia (DPAU) Contributing Research Studies (CRS) platform: https://portal.dementiasplatform.com.au/crs-directory/lrgs-tua. Data can also be requested through the study team (Principal Investigator: suzana.shahar@ukm.edu.my).
16	Malaysia Ageing and Retirement Survey (MARS)	Malaysia	2018	Wave 2: 2021–22	5613 (Wave 1) 4497 (Wave 2)	Face-to-face interview (CAPI) Anthropometric measurements Blood pressure Physical performance measurements	40 years and above	57.2	39.8%	44.2%	No schooling (12.0%), Primary school (29.5%), Lower secondary (21.1%), Upper secondary (25.8%), Post-secondary/Tertiary education (11.6%)	Public Access Files can be accessed through https://swrc.um.edu.my/mars-data for both Waves 1 and 2, while microdata needs to be applied for through the MARS Data Application Form.
17	The Malaysian Cohort (TMC) project	Malaysia	2006–2012	Wave 2: started in 2013 Comprehensive follow-up is planned every 5 years	106,527 (Wave 1), not available for follow-up waves	Baseline: Face-to-face interview (CAPI) Anthropometric measurements Biospecimen collection Blood pressure Physical/clinical examinations	35–70 years	50.0	<28.5%	42.2%	Primary (26.6%), Secondary (45.6%), Tertiary (24.8%), Null (3.0%) (across all age groups)	Data is available for collaborators who adhere to specific guidelines listed in the project website: https://www.ukm.my/mycohort/ms/penyelidikan-kami/
18	Longitudinal Study of Aging and Health in the Philippines (LSAHP)	Philippines	2018	Wave 2: 2023	5985 (Wave 1) 4028 (Wave 2)	Face-to-face interview (CAPI) Anthropometric measurements Blood pressure Physical performance measurements	60 years and above	69.0	100.0%	40.3%	No schooling/elementary (35.9%), High school (44.5%), College (19.7%)	LSAHP data are publicly available by registering at the Baseline Data Request Portal, alongside a data user agreement and proposal template: https://www.drdf.org.ph/lsahp-baseline-data-request-portal/. The full reports for Wave 1 and 2 can be accessed through the DRDF and ERIA websites.
19	Panel on Health and Ageing of Singaporean Elderly (PHASE)	Singapore	2009	Wave 2: 2011–12 Wave 3: 2015	4990 (Wave 1) 3103 (Wave 2) 1574 (Wave 3)	Face-to-face interview (PAPI^b^) Anthropometric measurements Blood pressure Physical performance measurements	60 years and above	72.8	100.0%	45.8%	No formal education (30.8%), Primary school (36.4%), Secondary school (23.6%), Above secondary school (8.8%)	Researchers can request de-identified data from waves 1–3 for academic research purposes. The full dataset will not be shared; only a subset of the dataset, containing the variables that are relevant for answering the research question/s or investigating the research topic/s the researchers are interested in will be shared. Data will be shared upon request after filling up a data request form and signing of a letter of undertaking. More details can be found on the study website (https://www.duke-nus.edu.sg/care/research/dataset-codebook).
20	Singapore Longitudinal Aging Study (SLAS-I and –II)	Singapore	2003–2004	Wave 2: 2005–2007 Wave 3:2007–2009	2804 (Wave 1) 2844 (Wave 2)	Face-to-face interview (unspecified) Anthropometric measurements Biospecimen collection Blood pressure Physical/clinical examination Physical performance measurements	55 years and above	66.0	–	36.8%	No education (19.2%), Primary (33.1%), Secondary/higher (47.8%)	Data is available, for researchers who meet the criteria, upon requests from the Principal Investigator (pcmrhcm@nus.edu.sg).
21	Retirement and Health Study (RHS)	Singapore	2014	Wave 2: 2016–17 Wave 3: 2018–2019 Wave 4: 2020–2021 Wave 5: 2022–2023 Wave 6: 2024–2025	15,013 (Wave 1) 12,869 (Wave 2) 11,700 (Wave 3) 14,700 (Wave 4)	Face-to-face interview (unspecified) Online video call interview (since COVID)	45 years and above	–	49.5%	52.0%	No formal education (20.5%), Elementary school (18.0%), Middle school (37.3%), High school (5.2%), Vocational school (3.1%), Community college diploma (4.2%), Degree and above (11.5%)	The RHS website does not list any plans to make the dataset publicly available. The data is accessible only to government agencies or researchers working with/for government agencies. The RHS study team can be reached at cpf_rhs@cpf.gov.sg
22	Transitions in Health, Employment, Social Engagement, and Inter-generational Transfers in Singapore Study (THE SIGNS Study)	Singapore	2016–2017	Wave 2: 2019 Wave 3a: 2023–2024 Wave 3b: 2024–2025	4549 (Wave 1) 2887 (Wave 2) 1535 (Wave 3a) 5306 (Wave 3b)	Face-to-face interview (CAPI) Anthropometric measurements Blood pressure Physical performance measurements	60 years and above	70.9	100.0%	46.7%	No formal education (27.5%), Primary (30.6%), Secondary (29.2%), Tertiary (12.6%)	Currently, de-identified data is available only for collaborators of the study team. The full dataset will not be shared; only a subset of the dataset, containing the variables that are relevant for answering the research question/s or investigating the research topic/s the researchers are interested in will be shared. Data will be shared upon request after filling up a data request form and signing of a letter of undertaking (care-datarequest@duke-nus.edu.sg), subject to approval from the study stakeholders and funder.
23	Korean Frailty and Aging Cohort Study (KFACS)	South Korea	2016–2017	Wave 2: 2018–2019 Plans for follow-up every 2 years, over a 10-year period	3014 (Wave 1) 2696 (Wave 2)	*Baseline:* Face-to-face interview (unspecified) Anthropometric measurements Biospecimen collection Blood pressure Physical/clinical examination Physical performance measurement *Follow-up:* Face-to-face interview (unspecified) Telephone interview	70–84 years	76.0	100.0%	47.5%	Less than middle school (48.2%), Middle and high school (34.8%), College and above (17%)	Data are available upon reasonable request. All data relevant to the study are included in the article or uploaded as supplementary information. All published articles and news articles using the KFACS database, data provision manuals and contact information are available at the KFACS website (http://www.kfacs.kr). The KFACS cohort database and blood samples are available to researchers, and the authors anticipate collaboration even with international researchers, although approval from the Kyung Hee University Hospital IRB is required to share the dataset or banked blood samples for all the researchers.
24	Korean Genome and Epidemiology Study_Health Examinee Study (KoGES HEXA)	South Korea	2004–2013	Wave 2: 2007—ongoing	173,357 (Wave 1)	*Baseline:* Anthropometric measurements Biospecimen collection Blood pressure Physical/clinical examination Physical performance measurement Face-to-face interview (unspecified) *Follow-up:* Mail survey Telephone interview	≥40 years	53.1	24.7%	34.2%	Education duration ≤6 years: 17.8% 7–12 years: 52.5% 12 ≤ years: 26.7%	Researchers can access the dataset after receiving approval from a designated research proposal review committee of the NIH. The genotype (genome-wide SNP data) and epidemiological dataset are made available to researchers after completing the quality control process. Sample sharing is restricted to genomic DNA among the archived biological materials for future use. Further information can be found on the National Institute of Health's website (https://nih.go.kr/ko/main/contents.do?menuNo=300919) or by email to the study team (kimye@korea.kr).
25	Korean Longitudinal Study of Aging (KLoSA)	South Korea	2006	Wave 2: 2008 Wave 3: 2010 Wave 4: 2012 Wave 5: 2014 Wave 6: 2016 Wave 7: 2018 Wave 8: 2020 Wave 9: 2022	10,254 (Wave 1) 8875 (Wave 2) 8229 (Wave 3) 7813 (Wave 4) 8387 (Wave 5) 7893 (Wave 6) 7491 (Wave 7) 7000 (Wave 8) 6592 (Wave 9)	Face-to-face interview (CAPI) Physical performance measurement	45 years and above	61.7	54.1%	43.5%	Middle school diploma or lower (62.5%), High school diploma (26.4%), Bachelor's degree or higher (11.2%)	KLoSA data, questionnaires, Coding Guide and other information are available through the website (https://survey.keis.or.kr/eng/klosa/klosa01.jsp) after validation and final review; Codebook available for download without logging in.
26	Taiwan Longitudinal Study on Aging (TLSA)^e^	Taiwan	1989	Wave 2: 1993 Wave 3: 1996 Wave 4: 1999 Wave 5: 2003 Wave 6: 2007 Wave 7: 2011 Wave 8: 2015 Wave 9: 2019 Wave 10: 2023	4049 (Wave 1) 3155 (Wave 2) 5131 (Wave 3) 4620 (Wave 4) 5377 (Wave 5) 4534 (Wave 6) 3727 (Wave 7) 8300 (Wave 8) 6490 (Wave 9) 5257 (Wave 10)	Face-to-face interview (PAPI before 2019, CAPI since 2019) Anthropometric measurements Biospecimen collection^d^ Blood pressure^d^ Physical/clinical examination^d^ Physical performance measurements^d^	60 years and above (refreshed samples are 50 and above)	67.6	100.0% (before refreshed sample)	51.5%	–	Due to legal restrictions imposed by the government of Taiwan in relation to the Personal Information Protection Act, data cannot be made publicly available. The use of TLSA data is limited to research purposes only. Requests for data can be sent as a formal proposal to the HWDC, Ministry of Health and Welfare (http://www.mohw.gov.tw/CHT/Ministry/Index.aspx), Taiwan and will be available with the permission of HWDC.
27	The Healthy Aging Longitudinal Study in Taiwan (HALST)	Taiwan	2009–2013	Wave 2: 2014–2019 Wave 3: Ongoing (started in 2020)	5664 (Wave 1) 4232 (Wave 2)	*Baseline:* Face-to-face interview (unspecified) Anthropometric measurements Biospecimen collection Blood pressure Physical/clinical examination Physical performance measurements *Follow-up:* Telephone interview	55 years and above	29.8% aged 55–64 years, 44.1% aged 65–74 years, 26.1% aged 75 years and above	70.0% (65 years and above)	47.2%	No formal education (14.1%), Elementary school (41.0%), High school (28.8%), University (16.1%)	The HALST study group encourages domestic and international research collaboration. To learn more about the HALST study, access the data and explore potential collaboration, please contact the principal investigator, Dr. Chao A. Hsiung (hsiung@nhri.org.tw).
28	Health, Aging, and Retirement in Thailand (HART)	Thailand	2014	Wave 2: 2017 Wave 3: 2020 Wave 4: 2022 Wave 5: 2024	5616 (Wave 1) 3708 (Wave 2) 3158 (Wave 3) 5629 (Wave 4), not publicly available for wave 5	Face-to-face interview (PAPI in wave 1 and CAPI from wave 2 onwards)	45 years and above	66.4	68.2%	47.8%	No formal education (6.8%), Primary education (70.3%), Secondary education (12.0%), Higher education (10.9%)	Data are available to be requested through the Download Center of the HART NIDA website: https://hart.nida.ac.th/download-center/. Login/Registration is required to access de-identified public datasets. Resources are mostly available in Thai; the codebook has been made available in English. Other guidelines can be found on the website: https://hart.nida.ac.th/data-product/.
29	Longitudinal Study of Ageing and Health in Vietnam (LSAHV)	Vietnam	2018	Wave 2: 2022–2023	5793 (Wave 1), not publicly available for wave 2	Face-to-face interview (CAPI) Anthropometric measurements Blood pressure Physical performance measurements	60 years and above	70.6	100.0%	42.8%	No schooling/Pre-school (20.8%), Elementary school (35.7%), Secondary school (22.4%), High school (9.6%), Vocational school (5.1%), College or higher (6.5%)	A baseline data request portal is available through the Institute of Population, Health and Development website alongside a data user agreement form and proposal template (https://www.phad.org/en/data-release/).
30	Vietnam Health and Aging Study (VHAS)	Vietnam	2018	Wave 2: 2021–2022	2447 (Wave 1), not publicly available for wave 2	Face-to-face interview (CAPI) Anthropometric measurements Biospecimen collection Blood pressure Physical performance measurements	60 years and above	–	100.0%	48.8%	None (9.9%), Primary (23.8%), Junior Secondary (46.4%), Senior Secondary (9.1%), Professional/Technical School (6.6%), College/University (4.3%)	Data can be requested from the study team upon the submission of a proposal, and all international publications utilizing the VHAS data must include at least one international principal investigator and one Vietnamese senior researcher as coauthors; please be sure that your application includes one of the VHAS co-investigators from Vietnam. Applications will be reviewed by lead VHAS personnel and a data access/approval decision will be provided within 10 working days of receipt of the application. More details can be found at: https://vhas.utah.edu/forms/data-use-proposal.php

a Unspecified: mode of face-to-face interview not publicly available.

b CAPI: Computer assisted personal interviewing; PAPI: Pen-and-Paper Personal Interview.

c Baseline statistics for study were not publicly available following the authors' search.

d Measured in Social Environment and Biomarkers of Aging Study (SEBAS), an ancillary study of Taiwan Longitudinal Study on Aging (TLSA).

e Also known as the Health and Living Status of the Elderly in Taiwan (HLSET) or Survey of Health and Living Status of the Near-elderly and Elderly in Taiwan or Survey of Health and Living Status of the Middle Aged and Elderly in Taiwan (SHLS).

Among the 28 studies which used face-to-face interviews, we were able to determine the modality adopted for data capture for 14 studies—most (n = 13) used computer assisted personal interviewing (CAPI) in one or more waves, with only one study using pen-and-paper personal interview (PAPI) across all waves. The earliest use of CAPI was in 2006 while PAPI was used even in 2018. In two out of the 13 studies employing CAPI, there was a shift from PAPI to CAPI across the waves and CAPI has now become the predominant modality for data capture for face-to-face interviews. The most recent longitudinal studies that started in 2010 onwards have demonstrated greater technological integration, with approximately 62% (n = 8 out of 13) using CAPI.

Administrative data linkage to conduct follow up among study participants was employed in three studies. Specifically, the Department of Health Elderly Health Service Cohort in Hong Kong SAR,^24^ the JPHC Study,^25^ and the Japan Public Health Center-based Prospective Study for the Next Generation (JPHC-NEXT).^50^

### Study population and sample size

We observed considerable variability across the included longitudinal studies regarding age eligibility, at baseline, of study participants (Table 1). While 11 studies only included adults aged 60 years and above, four studies only included adults aged 65 years and above. Another six studies applied a broader age range that included adults 40 or 45 years and above, and two additional studies included participants aged 55 years and above. One study specifically targeted those aged 80 years and above. The remaining six studies focused on adults within specific age ranges (e.g., aged 35–70 years, 50–75 years, 70–84 years).

Baseline sample sizes also varied across the longitudinal studies, with smaller studies such as the Longitudinal Study on Neuroprotective Model for Healthy Longevity (LRGS TUA, n = 2322),^41^ and the Healthy Ageing and Biomarkers Cohort Study (HABCS, n = 1462) including approximately 1000–2500 participants,^36^ while some large sample sizes exceeded 66,000 participants. Some of the large-scale studies include the Retirement and Health Study (RHS) in Singapore (n = 15,013),^49^ the China Health and Retirement Longitudinal Study (CHARLS, n = 17,697),^38^ the Department of Health Elderly Health Service Cohort in Hong Kong SAR (n = 66,820),^24^ the Malaysian Cohort project (n = 106,527),^31^ and the JPHC Study (Japan, n = 113,461).^25^ Within each study, although sample sizes (from baseline) decreased over time due to mortality and loss to follow up, all studies had greater than 1000 participants at the most recent wave of data collection.

### Key measurement constructs

Most studies collected comprehensive information on participants’ sociodemographics, such as gender, education level, and household composition. Table 2 presents the key health-specific constructs that were assessed in the 30 studies.

**Table 2:** Key health-specific constructs assessed in included studies (n = 30).

Note: Based on a search of available study questionnaires, scientific publications, and other published online materials.

1 Biospecimens, blood pressure and physical performance data were measured in Social Environment and Biomarkers of Aging Study (SEBAS), an ancillary study of Taiwan Longitudinal Study on Aging (TLSA).

All studies assessed one or more physical health constructs, measured using either questions, scales, or objective measurement. Most studies included questions or scales for assessing functional status in terms of ADLs (n = 27), IADLs (n = 23), and physical function (e.g., Washington Group Short Set on Functioning, “Nagi” functional limitations; n = 23). Many of the studies used objective measures to assess participants’ physical performance (e.g., Short Physical Performance Battery, handgrip strength, gait speed; n = 19) and incorporated questions or scales for physical activity (e.g., Global Physical Activity Questionnaire, International Physical Activity Questionnaire; n = 25). Only 12 studies included physical frailty measures.

Most studies (n = 26) assessed cognitive function of their participants through scales such as Mini-Mental State Examination. All studies, except for one, assessed one or more psychosocial health constructs, with social connectedness (e.g., social networks, loneliness, social participation etc.; n = 28) being the most widely measured construct, followed by depression or depressive symptoms (n = 26) and quality of life (n = 19). Measures of health literacy were included in 16 (53%) studies. About 90% of the studies (n = 27) incorporated anthropometric data such as height, weight, body mass index (either measured or self-reported), more than two-thirds (70%; n = 21) measured blood pressure, and half (50%; n = 15) collected biospecimens (such as blood and urine) for laboratory analysis. It should be noted that several studies used modified or contextualized versions of the same scale or measure to assess specific health constructs in their setting; thus, we do not provide citations of specific scales or measures and encourage researchers to go through specific study documentation for more information.

### Geographic representation

We observed uneven geographic distribution of longitudinal aging studies across ESEA. Specifically, high-income economies (Hong Kong SAR; Japan; Singapore; South Korea; and Taiwan) collectively accounted for 17 of the 30 longitudinal studies (57%). Mainland China, with the largest absolute number of older adults in the region, was represented by six studies. Although the aging population is increasing rapidly in the Southeast Asian region, only the Philippines, Singapore, Thailand, and Vietnam had established longitudinal aging studies. Of note, we did not find any eligible longitudinal aging studies from Brunei, Cambodia, Indonesia, Laos, Macao SAR, Mongolia, Myanmar, North Korea, or Timor-Leste.

### Data access and sharing policies

Across all the included studies, specific data access terms and conditions varied according to factors such as the intended use of study data and (academic) affiliation of requestors (Table 1). For example, several studies such as the LSMOP, CHARLS, China Longitudinal Aging Social Survey Study (CLASS), and Korean Longitudinal Study of Ageing (KLoSA) have included a portal on their websites that allows researchers to register and download their data directly. Other studies such as the Health Aging, and Retirement in Thailand (HART), Longitudinal Study of Ageing and Health in the Philippines (LSAHP), Malaysia Ageing and Retirement Survey (MARS), Panel on Health and Ageing of Singaporean Elderly (PHASE), TLSA, and Vietnam Health and Ageing Study (VHAS), albeit being publicly accessible, have more controlled access policies, such as requiring formal applications and/or research proposals, establishing an institutional-level data use agreement, or collaboration with study investigators to access data.

## Discussion

Our mapping of representative community-based health-focused longitudinal aging studies in ESEA identified 30 studies and revealed significant heterogeneity in data collection methods and measured health constructs, with substantial methodological evolution over time and uneven geographic distribution across the region. In doing so, this review highlights critical research gaps and identifies opportunities for future research and policy.

The included longitudinal studies collectively represented 10 countries, regions, or territories, with most studies (74%) from Japan, Mainland China, Singapore and South Korea. The geographic distribution reflects existing socioeconomic disparities in ESEA. Specifically, we found that high-income economies in the region (Hong Kong SAR, Japan, Singapore, South Korea, and Taiwan) collectively accounted for 57% of the identified studies. It is possible that these higher-income countries, regions or territories have more established academic and public health research institutions, a more robust workforce in gerontology, geriatric medicine and epidemiology, and dedicated funding mechanisms, all of which better position them to design, initiate, and sustain longitudinal aging studies.

In contrast, the complete absence of eligible longitudinal aging studies from Brunei, Cambodia, Indonesia, Laos, Macao SAR, Mongolia, Myanmar, North Korea, and Timor-Leste represents a critical gap in our understanding of aging in these unique contexts, which needs to be addressed urgently. For example, the historical conflicts and displacement in Cambodia and Laos may have a long-term impact on their aging populations.^51^ The nomadic pastoral traditions in Mongolia may create unique aging experiences with seasonal migration and extreme geographic isolation that are not captured in any existing longitudinal studies.^52^ The absence of longitudinal data from these countries, regions, or territories limits our understanding on how these specific cultural, historical, and geographic contexts shape aging trajectories as well as the development of culturally appropriate interventions tailored to their unique demographic challenges. While the baseline wave of planned longitudinal studies in some of these countries, regions or territories were identified in our search—for instance, The Healthy and Active Ageing in Myanmar (JAGES in Myanmar 2018),^53^ the Indonesia Longitudinal Aging Survey (ILAS),^54^ and the WHO Study on Global AGEing and Adult Health (SAGE)–China^55^—they were excluded as no details on any follow-up waves were publicly available.

Longitudinal aging studies in countries, regions, or territories where they are currently lacking can be initiated in collaboration with institutions and researchers from other settings, who are well experienced in the design, conduct and analysis of such studies. However, in doing so, avoidance of parachute science/research, and instead, the establishment of research collaborations that maintain inclusivity and equity, in the context of areas such as knowledge transfer, data ownership, authorship on research outputs etc., should be paramount.^56^ An example of an inclusive collaboration is the conduct of the LSAHP^48^ and LSAHV^46^ in the Philippines and Vietnam, respectively. These studies, funded by the Economic Research Institute for ASEAN and East Asia (ERIA; an international organization headquartered in Indonesia), are conducted by a team of ‘local’ researchers (led by a senior researcher) within the country, with experienced researchers from Japan serving as co-principal investigators. Furthermore, the study reports are jointly co-authored by the local and international investigators and institutions, study publications reflect the strong involvement of the local investigators, and sharing of the study data is handled by the local investigators. At the same time, while such collaborations can train the researchers who are directly involved, it is equally important to develop research capacity, at the level of both institutions and individual researchers, in relevant fields such as gerontology, geriatric medicine and epidemiology, more widely in these countries, regions, or territories. A situational analysis of training programs in gerontology and geriatric medicine in Southeast (and South) Asian countries, published in 2020, highlighted that such programs were either absent or limited in availability in several countries.^57^ Development of local capacity in aging research will ensure that the longitudinal data collected in these countries, regions, or territories is context-specific, addresses the practice and policy needs, as well as sustain a healthy pipeline of local researchers. While provision of detailed recommendations for local research capacity building is beyond the scope of this paper, they can range, in the short-term, from the research placement of local researchers with experienced international researchers to international scholarships for graduate or doctoral training, and in the long-term, the establishment of rigorous academic training programs focusing on gerontology, geriatric medicine and epidemiology within these countries, regions, or territories.

The temporal analysis of the included studies demonstrates a substantial expansion of health-focused longitudinal aging research between 2000 and 2019, suggesting increased recognition of the importance of longitudinal studies to understanding dynamic aging processes. However, we identified no new studies that were launched after 2020. Several factors may have contributed to this. First, the newly launched longitudinal studies might still be in the field and have not yet released their data for inclusion in our review or only finished their baseline wave. Additionally, the COVID-19 pandemic likely posed significant challenges to data collection efforts, potentially contributing to the postponement or suspension of both new research initiatives and ongoing data collection activities.^58^ Besides these challenges, the COVID-19 pandemic has also posed opportunities to advance aging research in several ways. First, it accelerated the adoption of digital technologies and remote monitoring in aging research, demonstrating both the feasibility and necessity of incorporating telemedicine, wearable devices, and smartphone-based data collection during public health emergencies.^59^^,^^60^ In addition, as the COVID-19 pandemic disproportionally affected older adults’ health and well-being,^61^ it has generated new research priorities such as the long-term physical and cognitive consequences of COVID-19 infection among older adults, the impact of prolonged social isolation on psychosocial well-being, and disrupted caregiving arrangements.^62^, ^63^, ^64^ Therefore, data from both new and existing longitudinal studies are needed to characterize the aging processes before and after the COVID-19 pandemic.

Our findings also indicate that data collection methods have gotten increasingly digitalized over the past four decades, with more recent studies using CAPI during face-to-face interviews and older cohorts switching from PAPI to CAPI during such interviews. Furthermore, more recent studies are more likely to incorporate biospecimen collection, physical performance measurement, and physical clinical examinations. This methodological evolution reflects a positive trend toward a more comprehensive and objective assessment of aging processes. However, important gaps remain in current data collection methods. First, there is limited integration of digital health technologies and passive data collection (e.g., data from wearable devices or sensors) in the current longitudinal studies. Such digital biomarker data can provide continuous health information that captures important changes in older adults’ health status between traditional data collection waves. Several ESEA countries, regions or territories such as Japan, Singapore, South Korea, and Taiwan are among the global leaders in digital health innovation and have demonstrated strong capacity in developing and deploying digital health technologies in both clinical and research contexts.^65^, ^66^, ^67^ Therefore, leveraging these regional strengths through targeted integration of emerging digital technologies into the design of future and existing longitudinal aging studies, in the short-to mid-term, could substantially improve data quality, completeness, and the depth of health information captured in these cohorts. Second, relative to objective health data collected through the conduct of anthropometric measurement (n = 22 studies), blood pressure (n = 20) and physical performance measurement (n = 19), genetic or biomarker data through the collection of biospecimens remains less common (50%; n = 15 studies). As the molecular and genetic determinants of healthy aging in Asian populations may differ significantly from the more extensively studied cohorts from North American and European countries, future studies would benefit from incorporating these technologies in the short-to mid-term to capture more granular, continuous, and biologically informative data such as the Taiwan Biobank^68^^,^^69^ to better characterize aging trajectories in the region.

Prior research has demonstrated that the aging processes are multi-dimensional and are shaped not only by individual characteristics but also by contextual factors at the community and societal levels.^70^^,^^71^ The integration of such multilevel data sources into existing longitudinal studies enables more sophisticated analyses of how environmental and social factors interact with individual characteristics to influence older adults’ health and well-being over time.^72^, ^73^, ^74^ While we did not make a systematic effort to assesses if all included studies collected contextual environmental and social data, we observed that certain studies did gather such data. For instance, the CHARLS incorporated a community survey to assess available community resources and assets.^38^ Deliberate incorporation of such community-level information will have great potential to inform more holistic health and social welfare policies that address both individual and contextual determinants of health in later life.

Significant gaps exist in the measurement domains across the included studies. Only slightly more than one-third of the studies included measures on physical frailty despite its growing recognition as a key predictor of adverse outcomes in later life.^75^ Similarly, only half of the studies included health literacy indicators and about two-third included quality of life indicators. These measurement gaps highlight the need for more comprehensive, person-centered measures that capture the multidimensional nature of aging experiences in future longitudinal studies.

Furthermore, previous studies have demonstrated the importance of informal care in many Asian contexts where family-based caregiving remains an essential support system for aging populations.^76^^,^^77^ Therefore, in addition to studying the aging process of older adults, it is critical to study informal caregivers caring for older adults to assess change in burden and benefits of caregiving over time, and characterize the health and well-being of caregivers. Although a methodical effort to assess whether all included studies collected data on informal caregivers of older adults was not made, we did observe relevant questions being asked in some of the studies. Examples include the Longitudinal Study of Aging and Health in the Philippines (LSAHP)^48^ and Longitudinal Study of Ageing and Health in Vietnam (LSAHV) which included a separate set of questions that were specifically asked to caregivers or potential caregivers of older adults. These existing parallel cohort studies, along with the development of new longitudinal studies that incorporate both care recipient and caregiver perspectives, could provide a robust framework for advancing our understanding of the complex interplay between aging processes and family-based caregiving systems in ESEA.

We also observed heterogeneity across studies in terms of the specific instruments used to measure common constructs such as cognitive function, physical function, depressive symptoms, quality of life, and social connectedness. Furthermore, different versions of the same instrument were used across studies, which likely reflects the need to contextualize item wordings, examples etc. to the study setting. While it is important to recognize and address culturally specific aspects of aging, establishing a common set of core measures for key constructs across studies would be critical for data harmonization and cross-national comparisons. There are ongoing efforts to harmonize measures across cohorts like the CHARLS, KLoSA, and MARS that are part of the Health and Retirement Study International Family of Studies.^78^ Still, not all identified cohorts participate in this initiative, and most harmonization has centered around sociodemographic and health domains. Therefore, future efforts, in the short-term, should focus on harmonizing measures in psychosocial domains, as well as emerging constructs like physical frailty, resilience, and digital literacy, while maintaining flexibility to capture contextual factors that contribute to the aging process.

Still, it is important to recognize that measurement differences across studies do not solely reflect inconsistency. These differences in measurements may represent deliberate and valuable adaptations to capture culturally specific dimensions of aging. The advances in psychometric methods, particularly item response theory, offer innovative approaches to allow data harmonization while preserving the local validity of original measures. A successful example of this approach is the Harmonized Cognitive Assessment Protocol, which has been implemented across multiple aging cohorts worldwide, including those from China and India.^79^ This example demonstrates that harmonization is an adaptive process in which measurement precision and cultural relevance can be preserved; similar approaches could be extended in the near future to other psychosocial and health constructs measured in ESEA longitudinal aging studies.

In addition to measurements, applying common data models (CDMs) could help address technical barriers such as data format heterogeneity and limited interoperability across longitudinal aging studies by standardizing the structure and content of data across studies. Use of CDMs, such as Observational Medical Outcomes Partnership (OMOP) CDM and Sentinel CDM, is common for standardizing electronic health record or clinical research data.^80^ While the direct application of such CDMs can be challenging for data measured in longitudinal aging studies, which often extends beyond clinical or physical health data to social, psychological, financial etc. data, there are examples of the application of such CDMs (largely, the OMOP CDM) to longitudinal population-based studies.^81^^,^^82^ Assessing which CDM is most suitable for longitudinal aging studies—not only in ESEA but also globally—such as can it accommodate the wide range of constructs, beyond clinical data, that are often measured in such studies, is beyond the scope of this study. However, the conduct of this assessment should be strongly considered in future research.

Built upon CDMs, it is critical to develop a regional and/or internal governance mechanisms to inform collaborative data-sharing. Since 2023, the National Institutes of Health (NIH) in the United States has implemented a Data Management and Sharing Policy that requires NIH-funded researchers to develop and implement a data management and sharing plan to maximize data accessibility and reuse while protecting participant privacy.^83^ Specifically, plans must describe the types of data generated, the standards and formats applied to facilitate interoperability, how and where data will be preserved and made accessible (including timelines for sharing), and what privacy protections or access limitations apply. Further, these plans are periodically reviewed and audited by the NIH to ensure compliance. A similar approach, through a Research Data Governance and Sharing Framework has been implemented by Singapore's National Medical Research Council (NMRC) since 2024.^84^ While these policies or frameworks applies specifically to NIH- or NMRC-funded research, respectively, they demonstrate how data governance frameworks can promote open-science and build shared data infrastructure at scale. For aging research in ESEA, analogous regional governance frameworks developed through multilateral bodies such as ASEAN, the WHO Western Pacific Region, or bilateral agreements between high-income and lower-income ESEA countries could establish shared standards for data management, access, and regulation. Such frameworks would need to address both technical requirements for data format compatibility and federated analytical infrastructure, as well as equity concerns described above.

Related, among the 30 identified longitudinal studies, the data access policies varied substantially. It might be that funding sources contribute to the availability and accessibility of data, as cohorts funded by large national and international organizations, such as the Health and Retirement Study International Family of Studies tend to establish more robust data-sharing infrastructures and make their data accessible to external researchers. Making data publicly available requires substantial efforts including data cleaning, curation, de-identification, and establishing appropriate governance frameworks to protect participant privacy while enabling scientific collaboration. Other studies have more controlled data-sharing policies that might be a barrier for external researchers to access their data, reflecting ongoing concerns about data ownership, quality control, and appropriate attribution. Addressing these regulatory and ethical issues around data sharing urgently will be critical to maximizing the scientific and policy value of existing longitudinal aging data resources in ESEA.

In addition to enhancing data sharing practices, there is a great need to expand existing and future longitudinal studies by establishing linkages with other data sources. While challenging to implement due to privacy regulations and technical barriers,^85^^,^^86^ linking such studies with electronic health records and/or administrative claims could substantially strengthen the utility of these studies. Some studies, such as the JPHC, JPHC-NEXT, and the Department of Health Elderly Health Service Cohort in Hong Kong SAR, already demonstrate successful integration with administrative health data and environmental data.^24^^,^^25^^,^^50^ In particular, countries, regions or territories such as South Korea and Taiwan have developed comprehensive national health insurance databases that offer substantial potential for enriching longitudinal aging research when linked with existing cohort studies. For instance, Taiwan's National Health Insurance Research Database and South Korea's National Health Insurance Service databases contain detailed longitudinal records of healthcare utilization, diagnoses, and initiation of new treatments and procedures across large populations.^87^, ^88^, ^89^ Another example is the establishment of the national-level ‘Trusted Research and Real World-Data Utilization and Sharing Tech’ (TRUST) platform in Singapore for secure linkage and analyses of anonymized research and administrative data at the individual level.^90^ Leveraging these resources through systematic linkage with community-based cohort studies could significantly address the identified gaps in health outcome data, enabling more comprehensive tracking of disease progression and healthcare service use among aging populations in ESEA. Furthermore, expanding these linkage approaches to include additional data sources—such as air quality data, social services databases, and economic indicators—could enable more comprehensive multilevel analyses of aging processes. The use of CDMs, mentioned before, across these varied data sources will be instrumental in facilitating data linkage.

Our review did not identify any existing longitudinal studies that included samples from multiple countries, regions or territories in ESEA. The success of multi-national studies like the Survey of Health, Ageing and Retirement in Europe (SHARE), which harmonizes data collection across 18 European countries, demonstrates the feasibility and value of coordinated cross-national aging research.^91^ Such multi-national approaches enable comparative analyses, pooled statistical power, and standardized methodologies that would be difficult to achieve through individual country, region or territory along. Multi-national studies could also serve as the foundation toward a broader regional consortium, facilitating knowledge sharing and methodological harmonization across diverse socio-economic contexts in Asia. The LSAHP^48^ and LSAHV^46^ demonstrate early success of coordinated, multi-country longitudinal studies that applied common data elements while maintaining country-specific measures. Thus, in the long-term, a similar consortium can be established and expanded across ESEA to address the limitations of current aging studies identified in this review, including heterogeneity in study design, restricted data access, and geographic imbalances in available data. Such an approach would require substantial coordination and investment but could yield significant benefits for understanding and addressing the challenges of population aging in the region.

At the same time, it is essential to acknowledge the substantial methodological, regulatory, and logistical challenges that such multi-national studies would entail. Specifically, ESEA has considerable political, economic, and cultural heterogeneity, and differences in national healthcare and long-term care systems, ranging from universal healthcare coverage models in countries, regions or territories like South Korea, Singapore and Taiwan to more fragmented systems elsewhere, complicate the cross-national comparability of healthcare utilization measures. Furthermore, obtaining ethical approval to allow cross-border data flow may require navigating multiple, and sometimes conflicting, institutional and national policies. Therefore, researchers may inevitably apply more conservative approaches to meet regulation requirements from multiple countries, regions or territories simultaneously. Such approaches may limit the scope of permissible data linkage and sharing. Taken together, addressing these challenges will require not only sustained cross-institutional commitment but also the development of region-specific data sharing and governance frameworks that are sensitive to the diverse legal, ethical, and operational contexts across ESEA.

This review has several limitations. First, our search strategy may not have captured all existing longitudinal aging studies in ESEA, particularly those with limited English-language documentation or those not indexed in the databases we searched. Second, variability in the access to the detailed documentation of study protocols and study design, and full data collection instruments (e.g., questionnaires, measurement instructions), led to the reliance on published reports or scientific papers for capturing some of the information presented in this paper, which may have led to some misclassification. Third, newly launched studies that have not yet completed their baseline data collection or only published initial findings may not have been identified in our search. Related, we recognize that some existing cohorts may have completed additional wave of follow-ups but are yet to release their most recent data. Such information may not have been included in our data extraction. Fourth, our review focused specifically on community-based longitudinal aging studies, which would have resulted in the exclusion of valuable clinical cohorts or cohorts of older adults living in long-term care facilities. Future reviews can focus on such cohorts to provide additional insights into the delivery and outcomes of clinical care or long-term care in ESEA. Fifth, although we applied inclusion criteria to ensure geographic diversity, it is important to acknowledge that longitudinal cohorts in this review may still be subject to attrition bias over time and may underrepresent hard-to-reach populations, such as rural and isolated older individuals, ethnic minorities, and the extremely poor. Lastly, we obtained information about data accessibility and sharing policies from publicly available sources, which may not reflect any informal data-sharing arrangements that exist between investigators.

## Conclusion

In this comprehensive database review, we identified, summarized, and compared the characteristics of 30 community-based health-focused longitudinal aging studies in ESEA over the past four decades. We identified several critical research gaps and proposed future directions including enhancing the representation of longitudinal aging studies in certain countries, regions or territories, establishing coordinated regional research initiatives, harmonizing core measures while maintaining cultural sensitivity, integrating digital health data, expanding biomarker and genetic data, incorporating caregiver assessments, improving data accessibility and linkage, and developing sustainable funding mechanisms. Addressing these gaps will be a key step to generate high-quality evidence that can inform policy making and promote healthy aging in ESEA.

## Contributors

Chenkai Wu conceived and designed the study, interpreted the results, and critically revised the manuscript. Rahul Malhotra and Hanzhang Xu interpreted the results, drafted the manuscript, and critically revised the manuscript. Haolin Li and Meagan Goh Yijing performed the literature review, extracted the data, conducted the analyses, and critically revised the manuscript. Leila Marie Magno, Huashuai Chen, Dararatt Anantanasuwong, Hideki Hashimoto, Zachary Zimmer, Halimah Awang, Yaohui Zhao, Vivian W.Q. Lou, Wenqian Xu, Yunhwan Lee, Lijing L. Yan, and Grace T. Cruz interpreted the results and critically revise the manuscript. All authors reviewed and approved the final manuscript. Rahul Malhotra, Hanzhang Xu, Haolin Li, Meagan Goh Yijing, and Chenkai Wu had full access to all the data in the study and had final responsibility for the decision to submit for publication.

## Declaration of interests

Hanzhang Xu was supported by the National Institutes of Health (R01AG089264, P30AG034424). Yunhwan Lee was supported by the National Research Foundation (NRF) grant funded by the Korean Government (Ministry of Science and ICT) (RS-2024-00350600). All other authors declare no competing interests.
