# Supplementary material for: Community-based health-focused longitudinal aging studies in East and Southeast Asia: landscape and future directions
Source: Lancet Reg Health West Pac. 2026 May 2;70:101867. doi: 10.1016/j.lanwpc.2026.101867 (PMC13221928; doi:10.1016/j.lanwpc.2026.101867)
Supplement: Supplementary Table S1 [file mmc1.docx]

**Supplementary Table 1.** Search strategy.

| **Data archive** | **Initial hits** | **Search strategy** |
| --- | --- | --- |
| PubMed | 907 | ("Singapore" OR "China" OR "Hong Kong" OR "Taiwan" OR "Macao" OR "Japan" OR "Mongolia" OR "Republic of Korea" OR "Brunei Darussalam" OR "Cambodia" OR "Indonesia" OR "Lao People's Democratic Republic" OR "Malaysia" OR "Myanmar" OR "Philippines" OR "Thailand" OR "Timor-Leste" OR "Vietnam" OR "Viet Nam") AND ("cohort profile" OR "longitudinal design" OR "national survey" OR "nationally representative survey" OR "longitudinal aging study" OR "longitudinal study of ageing") AND ("elderly" OR "retirement" OR "elder" OR "older adults" OR "ageing" OR "older people") AND ("2000/01/01"[Date - Publication] : "2024/12/31"[Date - Publication]) |
| International Journal of Epidemiology | 204 | Section:("Cohort Profile") AND (TEXT:("Elderly") OR TEXT:("Retirement") OR TEXT:("Elder") OR TEXT:("Older Adults") OR TEXT:("Ageing")) AND DATE:[2000-01 TO 2024-12] |
| BMJ Open | 319 | TITLE:("Cohort Profile") AND (TEXT:("Elderly") OR TEXT:("Retirement") OR TEXT:("Elder") OR TEXT:("Older Adults") OR TEXT:("Ageing")) AND DATE:[2000-01-01 TO 2024-12-31] |
| World Health Organization Multi-Country Studies Data Archive | 2 | ("elderly" OR "retirement" OR "elder" OR “older adults” OR "ageing") AND ("Singapore" OR "China" OR "Hong Kong" OR "Taiwan" OR “Macao” OR "Japan" OR "Mongolia" OR "Republic of Korea" OR "Brunei Darussalam" OR "Cambodia" OR "Indonesia" OR "Lao People's Democratic Republic" OR "Malaysia" OR "Myanmar" OR "Philippines" OR "Thailand" OR "Timor-Leste" OR "Viet Nam") AND DATE: [2000-01-01 TO 2024-12-31] |
| Gateway to Global Aging Data | 16 | ("elderly" OR "retirement" OR "elder" OR “older adults” OR "ageing") AND ("Singapore" OR "China" OR "Hong Kong" OR "Taiwan" OR “Macao” OR "Japan" OR "Mongolia" OR "Republic of Korea" OR "Brunei Darussalam" OR "Cambodia" OR "Indonesia" OR "Lao People's Democratic Republic" OR "Malaysia" OR "Myanmar" OR "Philippines" OR "Thailand" OR "Timor-Leste" OR "Viet Nam") AND DATE: [2000-01-01 TO 2024-12-31] |
| Integrative Analysis of Longitudinal Studies on Aging (IALSA) | 9 | ("elderly" OR "retirement" OR "elder" OR “older adults” OR "ageing") AND ("Singapore" OR "China" OR "Hong Kong" OR "Taiwan" OR “Macao” OR "Japan" OR "Mongolia" OR "Republic of Korea" OR "Brunei Darussalam" OR "Cambodia" OR "Indonesia" OR "Lao People's Democratic Republic" OR "Malaysia" OR "Myanmar" OR "Philippines" OR "Thailand" OR "Timor-Leste" OR "Viet Nam") AND DATE: [2000-01-01 TO 2024-12-31] |
| National Archive of Computerized Data on Aging | 4 | ("elderly" OR "retirement" OR "elder" OR “older adults” OR "ageing") AND ("Singapore" OR "China" OR "Hong Kong" OR "Taiwan" OR “Macao” OR "Japan" OR "Mongolia" OR "Republic of Korea" OR "Brunei Darussalam" OR "Cambodia" OR "Indonesia" OR "Lao People's Democratic Republic" OR "Malaysia" OR "Myanmar" OR "Philippines" OR "Thailand" OR "Timor-Leste" OR "Viet Nam") AND DATE: [2000-01-01 TO 2024-12-31] |
