# Supplementary material for: Community-based health-focused longitudinal aging studies in East and Southeast Asia: landscape and future directions
Source: Lancet Reg Health West Pac. 2026 May 2;70:101867. doi: 10.1016/j.lanwpc.2026.101867 (PMC13221928; doi:10.1016/j.lanwpc.2026.101867)
Supplement: Supplementary Table S2 [file mmc2.docx]

**Supplementary Table 2.** Primary reason for exclusion.

| **Study name (Acronym)** | **Country/Region/Territory in East and Southeast Asia** | **Primary reason for exclusion** |
| --- | --- | --- |
| The Hong Kong Cardiovascular Risk Factor Prevalence Study (CRISPS) and the follow-up studies | Hong Kong Special Administrative Region (SAR) | Disease focused |
| The prospective study on Chinese elderly with multimorbidity in primary care in Hong Kong | Hong Kong Special Administrative Region (SAR) | Institutional sample |
| Atma Jaya Cognitive and Aging Research (AJCAR) | Indonesia | Disease focused |
| Basic health research survey (RISKEDAS) | Indonesia | Cross-sectional study |
| Indonesia Family Life Survey (IFLS) | Indonesia | Baseline mean participant age below 50 years |
| Indonesia Longitudinal Aging Study (ILAS) | Indonesia | Longitudinal study by design but only one wave has been conducted or data from only one wave is available |
| National socio-economic surveys (SUSENAS) | Indonesia | Cross-sectional study |
| Comprehensive Survey of Living Conditions in Japan (CSLC) | Japan | Cross-sectional study |
| Ganka-Ekigaku Network (GEN) | Japan | Disease focused |
| Hisayama Study (HS) | Japan | Limited geographical coverage |
| Integrated Longitudinal Studies on Ageing in Japan (ILSAJ) | Japan | Collation of individual longitudinal studies |
| Japan's National Institute for Longevity Sciences-Longitudinal Study of Aging (NILS-LSA) | Japan | Limited geographical coverage |
| Keio interhospital Cardiovascular Studies-atrial fibrillation (KiCS-AF) | Japan | Disease focused |
| Keys to Optimal Cognitive Aging (KOCOA) | Japan | Limited geographical coverage |
| Nagahama Cohort project | Japan | Limited geographical coverage |
| National Survey on Social Security and People’s Life | Japan | Cross-sectional study |
| Neuron to Environmental Impact across Generation (NEIGE) study | Japan | Limited geographical coverage |
| Rationale and design of the Resource Center for Health Science (RECHS) project | Japan | Limited geographical coverage |
| Research on Osteoarthritis / Osteoporosis Against Disability study | Japan | Disease focused |
| Survey of Midlife in Japan (MIDJA) | Japan | Limited geographical coverage |
| The Japanese Population-based Osteoporosis (JPOS) Cohort Study | Japan | Disease focused |
| The Sukagawa Study | Japan | Limited geographical coverage |
| Tokyo Centenarian Study (TCS) | Japan | Limited geographical coverage |
| Tsuruoka Metabolics Cohort Study | Japan | Limited geographical coverage |
| China Health and Nutrition Surveys (CHNS) | Mainland China | Baseline mean participant age below 50 years |
| Chinese Acute-on-Chronic Liver Failure (CATCH-LIFE) study | Mainland China | Disease focused |
| Chinese General Social Survey (CGSS) | Mainland China | Cross-sectional study |
| Chinese Social Survey (CSS) | Mainland China | Cross-sectional study |
| Longitudinal Study of Older People in Anhui Province | Mainland China | Limited geographical coverage |
| Research on Early Life and Aging Trends and Effects (RELATE): A Cross-National Study | Mainland China | Cross-sectional study |
| Study on global AGEing and adult health (SAGE) | Mainland China | Limited geographical coverage |
| The China Hainan Centenarian Cohort Study (CHCCS) | Mainland China | Limited geographical coverage |
| The China Multi-Ethnic Cohort (CMEC) study | Mainland China | Limited geographical coverage |
| The Guangzhou Biobank Cohort Study | Mainland China | Limited geographical coverage |
| The Henan Rural Cohort | Mainland China | Limited geographical coverage |
| the Lanxi Cohort study | Mainland China | Limited geographical coverage |
| the Liyang cohort study on chronic diseases and risk factors monitoring in China (Liyang Study) | Mainland China | Disease focused |
| the Nanjing Diabetes Cohort (NDC) | Mainland China | Disease focused |
| The National Colorectal Cancer Cohort (NCRCC) study | Mainland China | Disease focused |
| The Shanghai Aging Study (SAS) | Mainland China | Limited geographical coverage |
| The Shanghai Men’s Health Study | Mainland China | Limited geographical coverage |
| The Shanghai Suburban Adult Cohort and Biobank (SSACB) | Mainland China | Limited geographical coverage |
| The Xinjiang Multiethnic Cohort Study (XMC) | Mainland China | Limited geographical coverage |
| Thousand-Village Survey (TVS) | Mainland China | Cross-sectional study |
| Tongji Cardiovascular Health Study | Mainland China | Disease focused |
| West China Health and Aging Trend (WCHAT) | Mainland China | Limited geographical coverage |
| World Health Survey | Mainland China; Laos; Malaysia; Myanmar; Philippines; Viet Nam | Cross-sectional study |
| Wuwei Cohort | Mainland China | Disease focused |
| Malaysian Elders Longitudinal Research (MeLOR) | Malaysia | Limited geographical coverage |
| National Health and Morbidity Survey (NHMS) | Malaysia | Cross-sectional study |
| National survey of Determinants of Wellness among Older Malaysian: A Health Promotion Perspective | Malaysia | Cross-sectional study |
| National survey of Identifying Psychosocial and Identifying Economic Risk Factor of Cognitive Impairment among Elderly | Malaysia | Limited geographical coverage |
| National survey of Mental Health and Quality of Life of Older Malaysians | Malaysia | Cross-sectional study |
| National survey of Patterns of Social Relationships and Psychological Well-Being among Older Persons in Peninsular Malaysia | Malaysia | Cross-sectional study |
| NHMS Older Persons' survey | Malaysia | Cross-sectional study |
| Transforming Cognitive Frailty into Later-Life Self-Sufficiency (AGELESS) cohort | Malaysia | Disease focused |
| Healthy and active ageing in Myanmar (JAGES in Myanmar 2018) | Myanmar | Longitudinal study by design but only one wave has been conducted or data from only one wave is available |
| Marikina Memory and Aging Project (MMAP) | Philippines | Limited geographical coverage |
| Philippine Study on Aging | Philippines | Cross-sectional study |
| Diet and Healthy Aging (DaHA) study in Singapore | Singapore | Limited geographical coverage |
| National Survey of Senior Citizens in Singapore (NSSC) | Singapore | Cross-sectional study |
| Singapore Survey on Informal Caregiving (SSIC) | Singapore | Cross-sectional study |
| Social Isolation, Health, and Lifestyles Survey (SIHLS) | Singapore | Cross-sectional study (baseline wave of Panel on Health and Ageing of Singaporean Elderly, an included study) |
| The Singapore diabetic cohort study | Singapore | Disease focused |
| The Singapore Epidemiology of Eye Diseases study (SEED) | Singapore | Disease focused |
| Well-being of the Singapore Elderly (WiSE) study | Singapore | Cross-sectional study |
| Korea Health Panel Annual Data (KHPAD) | South Korea | Baseline mean participant age below 50 years |
| Korea National Health and Nutrition Examination Survey (KNHNES) | South Korea | Cross-sectional study |
| Korea Welfare Panel Study (KWPS) | South Korea | Baseline mean participant age below 50 years |
| Korean Community Health Survey (KCHS) | South Korea | Cross-sectional study |
| Korean Longitudinal Healthy Aging Study (KLHAS) | South Korea | Longitudinal study by design but only one wave has been conducted or data from only one wave is available |
| Korean Longitudinal Study on Cognitive Aging and Dementia (KLOSCAD) | South Korea | Disease focused |
| Korean National Survey on Elderly Living Conditions and Welfare Desire (KNSELCWD) | South Korea | Cross-sectional study |
| Korean Urban Rural Elderly (KURE) study | South Korea | Limited geographical coverage |
| Living Profiles of Older People Survey (LPOPS) | South Korea | Cross-sectional study |
| National health insurance service-senior (NHIS-senior) cohort in Korea | South Korea | Insurance claim-based sample |
| National Survey of Older Koreans (NSOK) | South Korea | Cross-sectional study |
| The Environmental-Pollution-Induced Neurological EFfects (EPINEF) study | South Korea | Limited geographical coverage |
| The National Health Insurance Service-National Health Screening Cohort (NHIS-HEALS) in Korea | South Korea | Insurance claim-based sample |
| Elderly Nutrition and Health Survey in Taiwan | Taiwan | Cross-sectional study |
| National Health Insurance Research Database (NHIRD) | Taiwan | Insurance claim-based sample |
| National Health Interview Survey (NHIS) in Taiwan | Taiwan | Cross-sectional study |
| Social Environment and Biomarkers of Aging Study (SEBAS) in Taiwan | Taiwan | Ancillary study of Taiwan Longitudinal Study on Aging (TLSA)^1^ |
| Taiwan Initiative for Geriatric Epidemiological Research (TIGER) | Taiwan | Limited geographical coverage |
| Taiwan National Physical Activity Survey (TNPAS) | Taiwan | Cross-sectional study |
| The Taiwan MJ Cohort | Taiwan | Baseline mean participant age below 50 years |
| National Survey of Older Persons in Thailand (SOPT) | Thailand | Cross-sectional study |
| Thai National Health Examination Survey (TNHES) | Thailand | Cross-sectional study |
| Vietnam Aging Survey (VNAS) | Vietnam | Cross-sectional study |
| Vietnam Household Living Standards Survey (VHLSS) | Vietnam | Not health focused |

^1^ Also known as the Health and Living Status of the Elderly in Taiwan (HLSET) or Survey of Health and Living Status of the Near-elderly and Elderly in Taiwan or Survey of Health and Living Status of the Middle Aged and Elderly in Taiwan (SHLS)
